# Limited musculoskeletal benefits of artificial gravity combined with cycling during bed rest: Results from the BRACE study

**DOI:** 10.1113/EP093145

**Published:** 2025-11-29

**Authors:** Mirko Mandić, Tommy R. Lundberg, Jean‐Pol Frippiat, Adam C. McDonnell, Igor B. Mekjavić, Marie‐Pierre Bareille, Rebecca Billette de Villemeur, Rodrigo Fernandez‐Gonzalo

**Affiliations:** ^1^ Department of Laboratory Medicine, Division of Clinical Physiology Karolinska Institutet Stockholm Sweden; ^2^ Unit of Clinical Physiology Karolinska University Hospital Stockholm Sweden; ^3^ Stress, Immunity, Pathogens Laboratory, SIMPA Université de Lorraine Vandœuvre‐lès‐Nancy France; ^4^ Aerospace Medicine and Rehabilitation Laboratory, School of Sport, Exercise and Rehabilitation, Faculty of Health and Wellbeing Northumbria University Newcastle upon Tyne UK; ^5^ Department of Automatics, Biocybernetics and Robotics Jožef Stefan Institute Ljubljana Slovenia; ^6^ Institute for Space Medicine and Physiology (MEDES) Toulouse France

**Keywords:** countermeasures, European Space Agency, fat infiltration, head‐down tilt bed rest, skeletal muscle

## Abstract

Prolonged exposure to microgravity, simulated via 6° head‐down tilt bed rest (HDT), induces musculoskeletal deconditioning and negatively impacts body composition. This study evaluated whether a combination of aerobic exercise with artificial gravity (AG) offers superior protection in comparison to exercise alone. Twenty‐four healthy male participants completed 60 days of HDT, randomized into control (C), exercise‐only (EX) and exercise with AG (EX‐AG) groups. Muscle volume, intramuscular fat, body composition and isokinetic strength were assessed via whole‐body MRI and isokinetic dynamometry. All groups experienced thigh fat‐free muscle volume loss: C (10.5% ± 2.6%), EX (6.9% ± 2.4%) and EX‐AG (4.3% ± 2.4%), with EX‐AG showing significantly less atrophy than C (*p *< 0.001). Compared with C, EX‐AG preserved more muscle in both anterior (*p*  <  0.001) and posterior (*p *< 0.05) compartments, whilst EX preserved more muscle only anteriorly (*p *< 0.05). The fat ratio increased more in C (8.9% ± 6.0%) compared with EX‐AG (−0.8% ± 3.8%; *p *< 0.05) but not EX (6.5% ± 9.8%). Muscle fat infiltration increased across all groups (C, 7.0% ± 3.7%; EX, 6.2% ± 4.3%; EX‐AG, 3.1% ± 4.7%) but was not different between groups (*p *> 0.05). Maximal isokinetic torque decreased in all groups over all measured angular velocities but was not different between groups (*p *> 0.05). This is the first study to investigate the combination of AG and exercise as a countermeasure to body composition changes induced by long‐term bed rest. We showed that EX‐AG provided partial protection against muscle atrophy and fat accumulation but did not outperform exercise alone in preserving muscle quality, strength or overall body composition.

## Abstract (highlights)

- **What is the central question of this study?**
  Does a combination of aerobic exercise with artificial gravity provide superior protection against skeletal muscle atrophy and unfavourable body composition changes during prolonged bed rest, in comparison to exercise alone?
- **What is the main finding and its importance?**
  Our results demonstrate that the combination of aerobic exercise and artificial gravity offers partial protection against bed rest‐induced muscle atrophy and fat accumulation. However, it does not confer additional benefits over exercise alone in preserving muscle quality, strength or overall body composition. These findings inform the design of efficient countermeasures for musculoskeletal health in microgravity analogues.

## 1 INTRODUCTION

Spaceflight causes significant physical deconditioning (Vico & Hargens, 2018), as does the ground‐based analogue of 6° head‐down tilt bed rest (Fernandez‐Gonzalo et al., 2024; Pavy‐Le Traon et al., 2007). These unloading conditions trigger physiological changes, including muscle atrophy (Alkner & Tesch, 2004; Narici et al., 2003), lumbopelvic deconditioning (Wesselink et al., 2025), bone density loss (Vico et al., 2000), reduced aerobic capacity (Capelli et al., 2006) and anaerobic capacity (Ferretti et al., 2001; Rittweger et al., 2007), and orthostatic intolerance (Blaber et al., 2011). These changes threaten the health and performance of astronauts.

As humanity progresses towards interplanetary travel, the level of space stressors that crews will face during extended space missions (i.e., microgravity, cosmic radiation and confinement) might not be addressed adequately by existing countermeasures (Fernandez‐Gonzalo et al., 2026). Although research on terrestrial spaceflight analogues and the International Space Station provide our current understanding of these challenges, deep space missions require improved countermeasure strategies to ensure mission success and crew safety (Liphardt et al., 2023). Despite this need for improved strategies, exercise is a fundamental spaceflight countermeasure with benefits across multiple physiological systems (Fernandez‐Gonzalo et al., 2026). However, mission constraints limit exercise time and space, and current exercise protocols cannot fully counteract the negative adaptations from prolonged microgravity exposure (Scott et al., 2023; Trappe et al., 2009) or ground‐based analogues (Alkner & Tesch, 2004). Therefore, combined interventions are of interest to maximize the benefits within these constraints.

Artificial gravity (AG) has been proposed as a promising countermeasure to address the physiological challenges of space travel (Clément et al., 2016). By generating centrifugal forces through rotation, AG simulates gravity effects and has shown potential in mitigating muscle atrophy (Caiozzo et al., 2009) and cardiovascular deconditioning (Linnarsson et al., 2015; Stenger et al., 2012). Unlike traditional countermeasures that target individual physiological systems, AG might offer a systemic approach, potentially addressing multiple issues simultaneously, for example, in the musculoskeletal and cardiovascular systems. This integrated solution is particularly important given the time and logistical constraints of implementing multiple countermeasures during missions.

Combining exercise with AG might provide synergistic benefits against physiological deconditioning during spaceflight (Smith et al., 2009; Tran et al., 2021). Although exercise protocols performed at the International Space Station, such as resistance and aerobic training, are somewhat effective in combating muscle atrophy, bone loss and cardiovascular deconditioning (Scott et al., 2023), integration with AG might amplify these benefits (Iwase, 2005; Iwase et al., 2004). AG provides a continuous gravitational stimulus complementing the intermittent nature of exercise, potentially enhancing muscle and bone adaptation whilst maintaining cardiovascular function and orthostatic tolerance. This approach addresses multiple physiological systems simultaneously and might improve the effectiveness of countermeasures for long‐duration space missions. It follows that a detailed analysis of the impact of the different combinations of AG with exercise on multiple organ systems is necessary.

In the present study, we investigated whether cycling exercise combined with AG using a short‐arm human centrifuge prevents or reduces the detrimental effects of 60 days of anti‐orthostatic bed rest on muscle volume, body composition and muscle strength. The secondary objective was to investigate whether supine cycling with AG is more effective than supine cycling alone in preventing or reducing the detrimental effects of head‐down tilt bed rest.

## 2 MATERIALS AND METHODS

### 2.1 Ethical approval

All subjects were informed verbally and in writing about the study before providing written informed consent to participate. The study conformed to the standards set by the *Declaration of Helsinki* and was approved by the ethical committee (Comité de Protection des Personnes Ile‐de‐France VI) on 19 December 2022 and by the French Drug Agency (ANSM) on 11 January 2023. In France, the study is registered under the ID‐RCB number 2022‐A02074‐39. The study is also registered in the Clinical Trials Database with the identifier: NCT06544213. The handling and analysis of data were also approved by the Swedish Ethical Review Authority (2022‐06455‐01).

### 2.2 General design

The present study is part of the Bed Rest with Artificial Gravity and Cycling Exercise (BRACE) project sponsored by the European Space Agency (ESA) and the French Space Agency (CNES). Twenty‐four healthy male volunteers were selected to participate in the study. Each subject stayed in the research facilities (MEDES Space Clinic, Toulouse, France) for 88 days, divided into three phases: (1) 14 days for baseline data collection (BDC‐14 to BDC‐1), in ambulatory conditions within a confined area, with standardized physical activity; (2) 60 days of strict bed rest with the head down at 6° (HDT1 to HDT60) with or without countermeasures; and (3) 14 days for post‐HDT assessments and recovery (R+0 to R+13), in ambulatory conditions in a confined area. All tests and measurements were conducted in a consistent order and standardized manner throughout the campaign. A detailed overview of the recruitment and selection process can be found in Appendix 1.

The participants were assigned randomly to one of three experimental groups, each group consisting of eight subjects (Table 1). No countermeasure protocol was performed in the control group (C). In the exercise‐only group (EX), supine cycling was used as a countermeasure. The third group, exercise in artificial gravity (EX‐AG), performed supine cycling whilst exposed to AG.

**TABLE 1:** Baseline characteristics of the participants (all male) in the control (C), exercise‐only (EX) and exercise in artificial gravity (EX‐AG) groups.

Characteristic	*C*	EX	EX‐AG
Age, years	29 ± 7	30 ± 5	30 ± 6
Height, cm	172 ± 7	178 ± 4	177 ± 7
Body mass index, kg/m^2^	24 ± 2	24 ± 2	24 ± 2
Peak oxygen uptake, mL/min/kg	45.5 ± 4.8	45.0 ± 3.7	44.9 ± 5.5
Artificial gravity level presyncope, Gz	1.41 ± 0.22	1.47 ± 0.17	1.46 ± 0.22

During the recovery phase, all participants took part in a rehabilitation programme based on the ESA Postflight Reconditioning Program (Petersen et al., 2017). This rehabilitation programme aimed to improve the recovery of subjects and help them towards regaining their pre‐study physical condition.

The BRACE study included multiple standard measures common to all bed rest studies supported by the ESA, with additional experiments from 14 research teams. Recently published data from BRACE showed that AG provided no additional benefit for preserving peak oxygen uptake (${\dot{V}}_{O_{2}\mathrm{peak}}$) or orthostatic tolerance compared with aerobic exercise alone (Hedge et al., 2025). Here, we report detailed assessments of body composition and phenotypic and functional skeletal muscle adaptations from the BRACE study.

### 2.3 Baseline data collection

During the BDC phase, two cardiopulmonary exercise tests were performed: One in the upright position and one in the supine position. Both tests followed exactly the same protocol, in which participants were asked to pedal at 50 W for 3 min at a minimum of 70 r.p.m., followed by an increase of 25 W/min until voluntary exhaustion. The data from the supine test were used to create the individual training programmes for the two groups with countermeasures (EX and EX‐AG). In brief, a linear regression between load (in watts) and O_2_ uptake was calculated from 75 W to peak load minus 25 W to determine the load on the cycle ergometer for the countermeasure as a percentage of ${\dot{V}}_{O_{2}\mathrm{peak}}$.

The orthostatic tolerance threshold (OT) and presyncope (PS) tests were performed in the ESA Short‐Arm Human Centrifuge (SAHC; MEDES). Participants wore a safety harness and were positioned in the supine position in the nacelle with their head towards the centre of the centrifuge. Research staff were monitoring the participants at all times from the control room during the tests, which included staff controlling the speed/acceleration (gravitational force in the *z*‐axis, Gz) of the centrifuge. Physiological monitoring included a five‐lead ECG to measure cardiac activity and non‐invasive photoplethysmography for continuous blood pressure measurement. Near‐infrared spectroscopy was used to assess regional blood oxygenation. Centrifugation started at 0.6 Gz (at the centre of gravity) for 10 min, followed by a stepwise increase of 0.1 Gz every 3 min until PS was achieved. PS was determined based on blood pressure, heart rate and subject‐reported symptoms. OT was defined as the Gz value causing a 20% increase in heart rate from baseline as determined by a quadratic fit of the heart rate‐response curve, and vasoconstriction as indicated by a significant and consistent decrease in oxygenated haemoglobin in the calf. Straining manoeuvres were monitored visually by observing the subjects’ legs, neck and face, and were controlled throughout centrifugation. Subjects were reminded before and during centrifugation to avoid moving or contracting their legs.

### 2.4 Magnetic resonance imaging

Fat distribution, skeletal muscle composition, and total lean and fat tissue volumes were assessed by MRI before (BDC‐9) and at day 52 of bed rest (HDT52). Participants underwent supine MRI using two‐point Dixon fat/water sequences on a 1.5 T scanner (Siemens Sola, Siemens Healthcare). Imaging parameters were as follows: 4–5 mm slice thickness, 6.62/6.63/15.74 ms repetition time, 2.39/4.60/4.77 ms echo time, 1 average, and 0.2560/0.4480/0.5120 pixels/mm resolution. The total scan time was <10 min. Fat and muscle quantification was performed using AMRA Researcher (AMRA Medical AB) (Borga et al., 2020; Karlsson et al., 2015; Mandić et al., 2020; West et al., 2018). MRI assessments were conducted prior to any muscle biopsy procedure and ≥36 h after any other study activity that could influence the outcome of the MRI assessment.

Following the acquisition of fat‐ and water‐separated images, the post‐processing of images involved four main steps: (1) calibration of fat images using fat‐referenced MRI; (2) generation of automatic segmentation through multi‐atlas registration of ground truth labels for fat and muscle compartments to the acquired images; (3) manual quality control by two independent, trained operators; and (4) quantification of fat, muscle and lean volumes, in addition to fat fraction and fat infiltration.

For muscle quantification, the sum of the voxels in the segmented muscle mask containing <50% fat was considered viable muscle tissue. The average proportion of fat in the viable muscle tissue was reported as muscle fat infiltration (MFI). More details about the software can be found in previous work (Borga et al., 2020; Karlsson et al., 2015; West et al., 2018).

The analysis was performed for all images simultaneously, and all segmented data were checked for visual quality by a trained operator prior to analysis. The following parameters were analysed: total lean tissue volume (TLT), visceral adipose tissue (VAT), abdominal subcutaneous adipose tissue (SAT), total thigh fat‐free muscle volume (FFMV), individual thigh muscle volumes (left/right, anterior/posterior), MFI, liver proton density fat fraction, total adipose tissue (TAT) and fat ratio (TAT/TLT). The total tissue volumes were quantified from the neck to the knee.

### 2.5 Isokinetic peak torque

The isokinetic peak torque of the knee extensors and flexors was assessed using isokinetic dynamometry (CON‐TREX AG, Dübendorf, Switzerland) at BDC‐4 and R+2. Prior to testing, participants completed a 10 min warm‐up on a cycle ergometer (50 W; 60–70 r.p.m.). They were then stabilized with straps securing the chest, hips and thighs, and the ankle lever arm was aligned with the axis of rotation of the knee joint. To complete the warm‐up, participants performed 10 consecutive submaximal extension–flexion repetitions at 30°/s. Maximal isokinetic knee extension and flexion torque was then measured at four angular velocities: 30, 90, 180 and 300°/s, with strong verbal encouragement. At each velocity, participants completed a submaximal set of three repetitions to familiarize themselves with the speed, followed by two maximal sets of three repetitions. There was a 1 min rest between sets at the same speed and a 2 min rest between sets at different speeds. The peak torque values for flexion and extension at each velocity were used for data analysis.

### 2.6 Countermeasures

**FIGURE 1:**
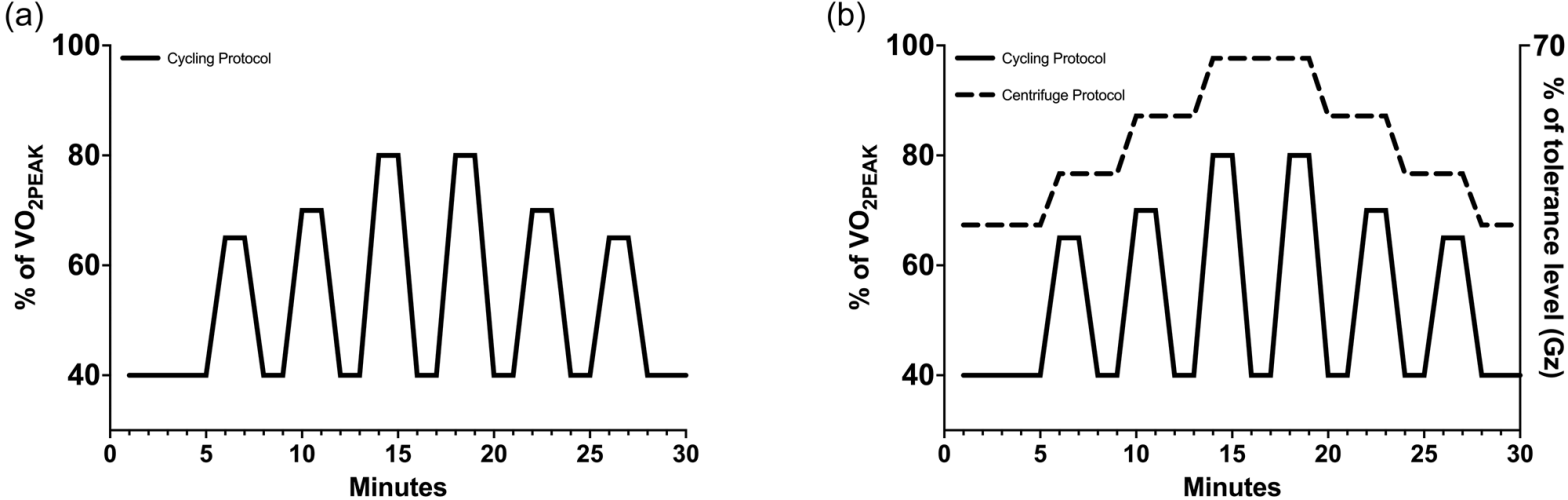
(a) Countermeasure protocol for exercise‐only group. (b) Countermeasure protocol for exercise and artificial gravity group. Abbreviation: ${\dot{V}}_{O_{2}\mathrm{peak}}$, peak oxygen uptake.

All exercise sessions, regardless of whether they were performed with or without AG, were performed according to the same protocol on the same cycle ergometer (Angio CPET, Lode) in the supine position with feet strapped to the pedals. Each session started at 40% of ${\dot{V}}_{O_{2}\mathrm{peak}}$ for 5 min, followed by six intervals of 2 min ranging from 65% to 80% of ${\dot{V}}_{O_{2}\mathrm{peak}}$. Intervals were interspersed with 2 min of low‐intensity cycling at 40% of ${\dot{V}}_{O_{2}\mathrm{peak}}$. Each session ended with a 3 min cool‐down period at 40% of ${\dot{V}}_{O_{2}\mathrm{peak}}$ (Figure 1a).

The centrifugation protocol was developed on the basis of the OT and PS tests described above. An individual protocol was created for each subject based on the individual Gz values corresponding to OT and PS (Goswami et al., 2015). The centrifuge started at 0.15 Gz below OT for 5 min; this is referred to as MIN (OT‐0.15). Every 4 min after this, Gz was increased by (MAX − MIN)/3 in synchronization with the cycle protocol up to 70% of the tolerance level of each subject; this is referred to as MAX [0.7(PS − OT) + OT]. Upon reaching 70% of the tolerance level, Gz was decreased by (MAX − MIN)/3 every 4 min until cessation of protocol (Figure 1b). The direction of rotation of the centrifuge was alternated between clockwise and anticlockwise at each session.

### 2.7 Statistics

Data were analysed using analysis of covariance (ANCOVA), with baseline values included as a covariate. The α‐level was set at 0.05 (*p* < 0.05), and Tukey's *post hoc* test was applied to evaluate differences between groups. All assumptions for ANCOVA were verified prior to analysis. Statistical analyses were performed using Jamovi (v.2.5.7.0). One subject was excluded from the isokinetic strength data owing to pain in the thigh in the BDC tests. One subject per group was excluded from the liver fat analysis because of movement during the scan. Unless otherwise stated, data are reported for 24 subjects. Results across time points are presented as the mean ± SD, and between‐group differences are reported as the mean difference, SE and 95% confidence intervals.

## 3 RESULTS

**FIGURE 2:**
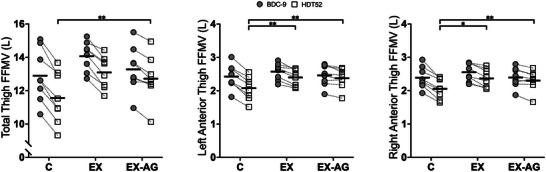
Changes in fat‐free muscle volume (FFMV) between baseline data collection −9 (BDC‐9) and head‐down tilt bed rest day 52 (HDT52) for control (C), exercise‐only (EX) and exercise in artificial gravity (EX‐AG) groups. Values are given for total thigh FFMV (left panel), left anterior thigh FFMV (central panel) and right anterior thigh FFMV (right panel) as group means (horizontal black line) and individual data with directional changes. *Post hoc* comparison: *p < 0.05 and **p < 0.001.

Total thigh FFMV decreased with the HDT intervention and varied significantly between groups (*p* < 0.001). The reductions were 10.5% ± 2.6% in C, 6.9% ± 2.4% in EX and 4.3% ± 2.4% in EX‐AG. The decrease in total thigh FFMV was significantly greater in C than in EX‐AG (*p* < 0.001). However, no other significant differences were found between the groups (Figure 2).

In the left leg, anterior FFMV decreased by 14.5% ± 3.7% in C, 6.8% ± 2.9% in EX and 3.5% ± 3.0% in EX‐AG. In both countermeasure groups, FFMV loss was significantly lower compared with C (*p *< 0.001). The difference between the two groups with countermeasures was not significant (*p *= 0.143). In the right leg, the anterior FFMV decreased by 14.0% ± 4.5% in C, 7.3% ± 3.2% in EX and 3.9% ± 4.2% in EX‐AG. In a similar manner to the left leg, the muscle loss was significantly larger in C compared with EX (*p *= 0.013) and EX‐AG (*p *< 0.001), without any significant difference between the two countermeasure groups (*p *= 0.263; Figure 2; Table 2).

**TABLE 2:** Magnetic resonance imaging variables measured and their differences between baseline data collection −9 (BDC‐9) and head‐down tilt bed rest at day 52 (HDT52).

	C			EX			EX‐AG		
Parameter	BDC‐9	HDT52	Percentage change	BDC‐9	HDT52	Percentage change	BDC‐9	HDT52	Percentage change
Weight, kg	71.7 ± 8.3	69.5 ± 8.6	−3.2 ± 1.1	75.7 ± 8.1	73.4 ± 7.1	−3.0 ± 1.7	73.6 ± 7.9	71.9 ± 7.7	−2.3 ± 1.4
Skeletal muscle									
Total thigh FFMV, L	12.9 ± 1.6	11.6 ± 1.5	−10.5 ± 2.6	14.1 ± 0.9	13.1 ± 1.0	−6.9 ± 2.4	13.3 ± 1.4	12.7 ± 1.4**	−4.3 ± 2.4
Left anterior thigh FFMV, L	2.4 ± 0.4	2.1 ± 0.4	−14.5 ± 3.7	2.6 ± 0.3	2.4 ± 0.3**	−6.8 ± 2.9	2.5 ± 0.3	2.4 ± 0.3**	−3.5 ± 3.0
Left posterior thigh FFMV, L	4.0 ± 0.5	3.7 ± 0.5	−8.0 ± 2.2	4.5 ± 0.2	4.2 ± 0.3	−6.9 ± 2.4	4.2 ± 0.5	4.0 ± 0.4*	−4.5 ± 2.4
Right Anterior thigh FFMV, L	2.4 ± 0.3	2.1 ± 0.3	−14.0 ± 4.5	2.6 ± 0.3	2.4 ± 0.3*	−7.3 ± 3.2	2.4 ± 0.3	2.3 ± 0.3**	−3.9 ± 4.2
Right posterior FFMV, L	4.1 ± 0.5	3.7 ± 0.5	−8.5 ± 2.2	4.5 ± 0.2	4.2 ± 0.2	−6.8 ± 2.6	4.2 ± 0.4	4.0 ± 0.4*	−4.6 ± 1.5
Mean thigh MFI, %	5.2 ± 1.7	5.5 ± 1.7	7.0 ± 3.7	4.9 ± 1.0	5.2 ± 1.1	6.2 ± 4.3	5.0 ± 0.9	5.1 ± 0.9	3.1 ± 4.7
Mean anterior MFI, %	4.0 ± 1.5	4.3 ± 1.5	9.6 ± 6.6	3.7 ± 0.8	3.9 ± 0.7	6.5 ± 7.1	3.8 ± 0.8	3.9 ± 0.7	3.4 ± 8.1
Mean posterior MFI, %	6.4 ± 1.9	6.8 ± 1.9	5.4 ± 2.3	6.1 ± 1.3	6.4 ± 1.4	6.0 ± 3.7	6.2 ± 1.1	6.4 ± 1.2	3.1 ± 3.9
Left anterior thigh MFI, %	4.0 ± 1.5	4.4 ± 1.5	10.5 ± 7.9	4.1 ± 0.7	4.2 ± 0.6	4.9 ± 10.7	4.1 ± 1.0	4.1 ± 0.8	3.4 ± 10.8
Left posterior thigh MFI, %	6.5 ± 2.0	6.9 ± 2.0	6.4 ± 3.3	6.3 ± 1.3	6.6 ± 1.3	4.7 ± 5.0	6.3 ± 1.0	6.5 ± 1.0	2.2 ± 6.2
Right anterior thigh MFI, %	3.9 ± 1.5	4.2 ± 1.5	8.6 ± 6.6	3.3 ± 0.9	3.6 ± 0.9	8.7 ± 7.8	3.4 ± 0.9	3.6 ± 0.9	4.6 ± 7.8
Right posterior thigh MFI, %	6.4 ± 1.8	6.7 ± 1.9	4.4 ± 2.5	5.8 ± 1.4	6.2 ± 1.5	7.6 ± 5.3	6.0 ± 1.2	6.3 ± 1.3	4.3 ± 2.0
Weight‐to‐muscle ratio, kg/L	5.6 ± 0.4	6.0 ± 0.4	8.2 ± 3.1	5.4 ± 0.3*	5.6 ± 0.4	4.3 ± 2.4	5.6 ± 0.6	5.7 ± 0.6**	2.0 ± 2.3
Fat tissue									
Fat ratio, %	28.9 ± 6.7	31.3 ± 7.0	8.9 ± 6.0	25.3 ± 7.1	26.6 ± 6.3	6.5 ± 9.8	27.4 ± 10.8	27.1 ± 10.8*	−0.8 ± 3.8
Total abdominal adipose tissue index, L/m^2^	1.8 ± 0.6	1.8 ± 0.6	1.0 ± 5.6	1.6 ± 0.6	1.6 ± 0.5	1.0 ± 12.2	1.7 ± 0.8	1.6 ± 0.8	−5.3 ± 3.8
Abdominal subcutaneous adipose tissue volume, L	3.7 ± 0.9	3.6 ± 0.9	−1.6 ± 6.0	3.4 ± 1.5	3.3 ± 1.3	−2.9 ± 10.0	3.5 ± 2.0	3.2 ± 1.9	−9.0 ± 3.9
Visceral adipose tissue volume, L	1.6 ± 0.8	1.7 ± 0.8	7.2 ± 6.7	1.6 ± 0.6	1.7 ± 0.6	10.3 ± 22.0	1.8 ± 0.8	1.8 ± 0.8	1.6 ± 5.3
Visceral adipose tissue index, L/m^2^	0.6 ± 0.3	0.6 ± 0.3	7.2 ± 6.7	0.5 ± 0.2	0.5 ± 0.2	10.3 ± 22.0	0.6 ± 0.2	0.6 ± 0.2	1.6 ± 5.3
Visceral adipose tissue ratio, %	29.2 ± 6.7	30.9 ± 7.0	6.2 ± 2.7	32.3 ± 7.5	34.8 ± 7.3	8.5 ± 7.9	34.4 ± 3.1	36.9 ± 3.8	7.3 ± 3.4
Liver fat, %	2.48 ± 2.45	2.94 ± 2.68	26.6 ± 14.8	1.52 ± 0.38	1.49 ± 0.61	4.6 ± 51.1	1.73 ± 0.72	1.95 ± 1.05	15.6 ± 33.3

*Note*: Abbreviations: C, control group; EX, exercise‐only group; EX‐AG, exercise in artificial gravity group; FFMV, fat‐free muscle volume; MFI, muscle fat infiltration. Liver fat, *n* = 21 (C = 7, EX = 7 and EX‐AG = 7). *Post hoc* comparisons compared with C: *p < 0.05 and **p < 0.001.

In all groups and both legs, posterior thigh FFMV decreased as a result of HDT. In the left leg, the decrease was 8.0% ± 2.2% in C, 6.9% ± 2.4% in EX and 4.5% ± 2.4% in EX‐AG. There were significant differences between groups (*p* = 0.031). The EX‐AG group lost significantly less FFMV compared with C (*p *= 0.041) but showed no difference when compared with EX (*p *= 0.100). There was no difference between EX and C (*p *= 0.913; Table 2). Posterior thigh FFMV in the right leg also decreased after HDT, with reductions of 8.5% ± 2.2% in C, 6.8% ± 2.6% in EX and 4.6% ± 1.5% in EX‐AG. There was a significant difference between groups (*p* = 0.013) driven by the difference between EX‐AG and C (*p *= 0.014). However, there were no significant differences between EX‐AG and EX (*p *= 0.093) or between EX and C (*p *= 0.726; Table 2).

**FIGURE 3:**
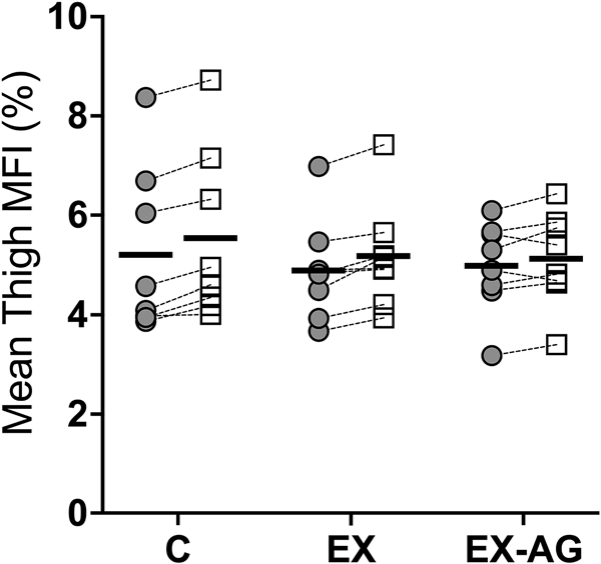
Changes in mean thigh muscle fat infiltration (MFI) between baseline data collection −9 (BDC‐9) and head‐down tilt bed rest day 52 (HDT52) for control (C), exercise‐only (EX) and exercise in artificial gravity (EX‐AG) groups, shown as group means (horizontal black line) and individual data with directional changes.

Mean thigh MFI increased after HDT in all groups, with no significant differences between groups (*p* = 0.165). In C, the increase was 7.0% ± 3.7%, in EX 6.2% ± 4.3% and in EX‐AG 3.1% ± 4.7%. Separating the analysis by leg (left, right) or by region (anterior, posterior) did not alter these results (Figure 3; Table 2).

Abdominal subcutaneous adipose tissue volume decreased in all groups as a result of HDT. The greatest decrease was seen in EX‐AG (−9.0% ± 3.9%), followed by EX (−2.9% ± 10.0%) and C (−1.6% ± 6.0%). These differences were not significant (*p* = 0.105). Total abdominal adipose tissue index increased in both C (1.0% ± 5.6%) and EX (1.0% ± 12.2%), whereas a decrease was seen in EX‐AG (−5.3% ± 3.8%). The differences between groups were, however, not significant (*p* = 0.098; Table 2). Visceral adipose tissue volume increased in all groups as a result of HDT, but with no difference between groups (*p* = 0.269). The same was true for the visceral adipose tissue index and visceral adipose tissue ratio (Table 2). Fat ratio increased by 8.9% ± 6.0% in C and by 6.5% ± 9.8% in EX and decreased by 0.8% ± 3.8% in EX‐AG. Fat ratio was significantly different between C and EX‐AG following HDT (*p *= 0.004). No further differences between the groups could be seen (Table 2). The weight‐to‐muscle ratio was significantly different between the groups after HDT (*p* < 0.001). The two countermeasure groups showed no difference between each other (*p *= 0.299), whilst the increase in C was significantly greater than in EX (*p *= 0.016) and EX‐AG (*p* < 0.001; Table 2). The change in liver fat did not show any differences between groups (*p* = 0.463; Table 2).

The strength assessment showed that, as a result of the HDT, all groups decreased their maximal isokinetic strength over all tested angular velocities. There were, however, no differences between groups (Table 3; Table S1).

**TABLE 3:** Isokinetic variables and the response to 6° head‐down tilt bed rest at baseline (BDC‐4) and after 60 days of bed rest (R+2).

	C			EX			EX‐AG		
Parameter	BDC‐4	R+2	Percentage change	BDC‐4	R+2	Percentage change	BDC‐4	R+2	Percentage change
Flexion, 30°/s, N m	122.7 ± 35.0	96.5 ± 21.0	−19.3 ± 13.6	129.0 ± 20.0	99.7 ± 31.3	−22.8 ± 21.5	123.2 ± 27.2	97.5 ± 21.8	−20.4 ± 10.2
Extension, 30°/s, N m	151.5 ± 32.4	118.4 ± 28.8	−19.9 ± 21.2	177.5 ± 40.4	140.3 ± 34.8	−19.0 ± 20.2	170.2 ± 32.5	128.6 ± 35.6	−25.0 ± 12.7
Flexion 90°/s, N m	110.4 ± 21.8	94.8 ± 21.2	−13.3 ± 16.2	116.9 ± 22.4	98.6 ± 26.4	−16.5 ± 14.3	103.3 ± 35.2	89.5 ± 24.0	−13.2 ± 10.9
Extension 90°/s, N m	144.4 ± 28.3	104.6 ± 27.2	−25.9 ± 20.3	152.2 ± 27.7	129.1 ± 39.1	−16.1 ± 20.9	145.3 ± 31.0	112.5 ± 36.3	−24.3 ± 16.1
Flexion, 180°/s, N m	99.7 ± 17.2	82.9 ± 19.3	−17.5 ± 8.2	99.9 ± 9.1	85.2 ± 19.9	−15.7 ± 14.4	87.8 ± 12.8	77.5 ± 21.2	−12.8 ± 20.2
Extension, 180°/s, N m	122.8 ± 23.4	93.7 ± 22.2	−22.4 ± 18.0	137.8 ± 16.7	113.8 ± 26.7	−18.3 ± 12.0	128.3 ± 20.3	103.5 ± 29.5	−24.4 ± 23.9
Flexion, 300°/s, N m	77.1 ± 12.9	70.6 ± 16.3	−8.6 ± 13.0	79.4 ± 9.6	68.8 ± 10.2	−13.4 ± 8.2	72.5 ± 7.1	68.4 ± 6.5	−4.9 ± 12.4
Extension, 300°/s, N m	99.6 ± 20.0	72.2 ± 22.2	−27.3 ± 17.4	108.0 ± 13.5	89.7 ± 16.5	−17.2 ± 8.9	97.8 ± 16.1	85.6 ± 19.5	−11.3 ± 23.4

*Note*: *n* = 23 (C = 7, EX = 8 and EX‐AG = 8).

Abbreviations: C, control group; EX, exercise‐only group; EX‐AG, exercise in artificial gravity group.

Detailed statistical outputs of the comparisons and analyses can be found in Table S1.

## 4 DISCUSSION

In this study, we investigated the combination of AG with aerobic exercise as a countermeasure to the detrimental effects of prolonged bed rest. The intervention led to reductions in muscle volume, increased muscle fat infiltration and negatively altered body composition. The combined countermeasure (EX‐AG) offered protection against muscle atrophy but showed limited benefits for muscle quality, strength and body composition in comparison to the control (C). Additionally, there were no significant differences between EX‐AG and exercise only (EX) across any of the measured variables.

The present bed rest protocol reduced muscle volume in all groups. However, the countermeasures provided some protection, with the intervention groups showing less atrophy than the control group. In particular, the EX‐AG group showed less atrophy than the control group both in the total thigh FFMV and in the segmented analysis of left, right, anterior and posterior muscles. It appears that the countermeasures had the greatest effect on the anterior quadriceps muscles, as evidenced by larger effect sizes (Table S1) and differences between EX and C in this segment only. However, there was no difference between the two countermeasure groups. These protective effects are on a par with those of other aerobic exercise modalities in combination with low volumes of resistance exercise (Krainski et al., 2014), but lag behind those of flywheel ergometer resistance training (Alkner & Tesch, 2004). The atrophy of the control group was in line with previous studies with similarly prolonged bed rest and supported the expected HDT‐induced atrophy (Miokovic et al., 2012; Mulder et al., 2009; Ploutz‐Snyder et al., 2018). These results suggest that aerobic exercise combined with AG offers protection when compared with no countermeasure at all, but it does not appear to be more effective when compared with exercise only.

We observed increased intramuscular fat infiltration (myosteatosis), indicating reduced muscle quality with prolonged simulated microgravity. Myosteatosis has previously been associated with inactivity (Belzunce et al., 2021; Manini et al., 2007) and various pathologies (Addison et al., 2014). The molecular signature for muscle fat accumulation is already evident after 3 days of reduced activity (Pagano et al., 2018) and originates from excessive influx of triacylglycerols from the bloodstream and/or from impaired muscle fat oxidation. Physical inactivity reduces plasma triacylglycerol uptake by downregulating lipoprotein lipase activity, suggesting that altered fat oxidation causes inactivity‐induced muscle fat accumulation (Bey & Hamilton, 2003; Zderic & Hamilton, 2006). In patients with chronic diseases, exercise reduces intramuscular fat with small effects but still associated with exercise‐induced loss of weight and fat mass (Tuñón‐Suárez et al., 2021). This was not the case in our young, healthy participants after prolonged HDT, because the countermeasures had very little effect on intramuscular fat infiltration. The differential effect of exercise on fat infiltration might be explained by the health status of the population investigated and potential divergences in the intensity and mode of exercise between studies. The EX‐AG group in our study maintained body fat percentage, whilst the controls increased. Consistent with this, the countermeasures showed no protective effect against negative shifts in other body composition measures (e.g. VAT, SAT, TAT and liver fat). On the positive side, both groups with countermeasures showed lower negative shifts in the weight‐to‐muscle ratio than the control group. However, the lack of global protection is of concern, because intramuscular fat infiltration and fat accumulation are associated with insulin resistance (Goodpaster et al., 1997, 2000) and impaired mobility (Visser et al., 2005).

Muscle function, measured as isokinetic torque at different angular velocities, decreased markedly in all groups, with no evidence of protection by countermeasures. In comparison to previous data from men, the decline was similar but in the lower range (Ploutz‐Snyder et al., 2018) and less than predicted by the model of Marusic et al. (2021). It is clear that any strategy aimed at counteracting the muscular deconditioning and loss of strength associated with space travel must include resistance exercise, because aerobic exercise with AG is not effective in this regard.

Previous studies using AG as a standalone countermeasure showed a protective effect against HDT‐induced deconditioning. In a 21 day HDT study, AG was reported to preserve knee‐extensor isokinetic torque, improve plantar flexor torque, maintain soleus muscle fibre cross‐sectional area and attenuate myostatin upregulation (Caiozzo et al., 2009). Additional benefits include improved vertical jump performance, a marker of lower‐limb function (Rittweger et al., 2015), which is closely associated with walking and stairclimbing performance (Runge et al., 2004). Given these results, the combination of AG with exercise should reasonably produce greater adaptation effects (Rittweger et al., 2015). Therefore, the lack of significant differences between the groups with countermeasures in our study is surprising, and it might be attributable to a longer duration of the bed rest period than previous studies or differences in the AG protocol (Caiozzo et al., 2009).

The study has several notable strengths. It was exceptionally well controlled. Participants’ diets, their baseline levels of physical activity and their individual exercise schedules were carefully monitored and strictly adhered to. This level of control increases the internal validity and strengthens the overall quality of the study.

Despite these strengths, several factors influence the interpretation of the results. The sample size of eight subjects per group is small, which is a common challenge in bed rest studies. As a result, only variables with very large effect sizes (Table S1) and low variability had sufficient statistical power. Although the data suggest possible additional effects of AG in some of the measured variables, we cannot draw definitive conclusions about their benefit. Future studies could use control groups derived from pooled data from several controlled bed rest studies instead of small concurrent control groups. In addition, there is a case for ambulatory control groups to examine individual variability to bed rest interventions (Fernandez‐Gonzalo et al., 2021). Allocating more participants to the intervention arms can significantly increase statistical power to detect changes and interactions with smaller but potentially meaningful effect sizes. Given the standardized protocols and controlled conditions in bed rest studies, harmonized control datasets provide reliable comparative references. This approach preserves scientific rigour whilst allowing a more comprehensive examination of hypotheses and countermeasures without the typical sample size limitations. These efforts should be conducted in parallel with investigating the effects of different exercise types (aerobic vs. resistance vs. combined) and modes of exercise and AG delivery (intermittent vs. continuous). Additionally, feasibility aspects relevant to real spaceflight conditions should be considered, including the size of countermeasure equipment, the time required for crew members to prepare and perform each countermeasure session, and the effectiveness of countermeasures in the hypocaloric conditions that typically occur during space missions (Fernandez‐Gonzalo et al., 2026).

## 5 CONCLUSION

In summary, although AG combined with aerobic exercise provided some protection against muscle wasting during prolonged bed rest, the effects on muscle quality, strength and body composition were modest. These findings emphasize the need to refine countermeasures, particularly to include some form of resistance exercise and optimize the intensity and mode of both exercise and AG, to effectively mitigate the musculoskeletal deconditioning associated with HDT. Although AG in combination with aerobic exercise offers limited musculoskeletal protection, it might still hold greater promise when combined with resistance exercise. This hypothesis will be explored in the upcoming Bed Rest with Artificial Gravity and Vibration and Resistance Exercise (BRAVE) study, supported by the ESA.

## AUTHOR CONTRIBUTIONS

Conception and design of the work (Jean‐Pol Frippiat, Adam C. McDonnell, Igor B. Mekjavić, Marie‐Pierre Bareille, Rebecca Billette de Villemeur, Rodrigo Fernandez‐Gonzalo); acquisition, analysis, or interpretation of data for the work (Mirko Mandić, Tommy R. Lundberg, Rebecca Billette de Villemeur, Rodrigo Fernandez‐Gonzalo); drafting the work or revising it critically for important intellectual content (Mirko Mandić, Tommy R. Lundberg, Jean‐Pol Frippiat, Adam C. McDonnell, Igor B. Mekjavić, Marie‐Pierre Bareille, Rebecca Billette de Villemeur, Rodrigo Fernandez‐Gonzalo). All authors approved the final version of the manuscript and agree to be accountable for all aspects of the work in ensuring that questions related to the accuracy or integrity of any part of the work are appropriately investigated and resolved. All persons designated as authors qualify for authorship, and all those who qualify for authorship are listed.

## CONFLICT OF INTEREST

The authors declare no conflicts of interest.

## Supporting information




**Appendix** **1**: Recruitment and selection of test subjects.

Detailed statistical output of comparisons and analyses

## Data Availability

All data supporting the findings of this study are available from the corresponding author (R.F.G.) on request.
