# Supplementary material for: Limited musculoskeletal benefits of artificial gravity combined with cycling during bed rest: Results from the BRACE study
Source: Exp Physiol. 2025 Nov 29;111(3):1181–90. doi: 10.1113/EP093145 (PMC12949100; doi:10.1113/EP093145)
Supplement: Supplementary file 1 — Appendix 1: Recruitment and selection of test subjects. [file EPH-111-1181-s002.docx]

Appendix 1. Recruitment and selection of test subjects.

The call for candidates was made via the Internet through the MEDES and ESA websites, as well as through various media channels (radio, social media, newspapers, etc.). The selection process was carried out in six phases:

1. **Initial expression of interest**Applicants had to send an email to MEDES to express their interest in participating in the study. A total of 3,193 candidates responded: 2,432 for the first campaign and 761 for the second. Based on the content of their emails, 150 people were excluded from the first campaign and 193 from the second campaign because they met one or more of the exclusion criteria. Due to the high number of applications, 1,767 people were placed on a waiting list, as MEDES was unable to process all submissions.
2. **Preliminary selection – via email**A primary selection was conducted via email with 1,083 applicants. This involved providing more detailed information about the study protocol, checking the main inclusion criteria, and clarifying any specific concerns. Across both campaigns, 828 candidates were either excluded during this phase or chose to drop out.
3. **Preliminary selection – via phone interviews**
   The next selection stage involved phone interviews with 149 candidates from the first campaign and 106 from the second. The interview included a discussion of the candidate’s lifestyle, education, professional experience, and a medical questionnaire covering personal and family history. Pre-selected volunteers received the information and consent document to allow them to make an informed decision. An investigator followed up with a dedicated call to explain the study in detail and answer any questions. This session aimed to clarify all aspects of participation and provide a reflection period. A physician also verified certain eligibility criteria (e.g., medical history, nutritional preferences, physical activity levels). After excluding 99 candidates from the first campaign and 60 from the second, 96 volunteers were invited to MEDES for an in-person selection visit to determine if they met the detailed eligibility criteria.
4. **Selection visit 1 - medical screening**
   Following 12 withdrawals, 84 candidates—42 from each campaign—underwent their first in-person selection visit. This visit included clinical and paramedical assessments with a focus on nutrition and physical activity. Tests included a DEXA scan (lumbar spine and left hip), ECG, stand test, urinalysis strip and urine drug tests, comprehensive bloodwork (including screening for factor II and V mutations), V̇O_2peak_ test, and a familiarization session with the centrifuge. This phase resulted in the exclusion of 30 subjects and six drop-outs.
5. **Selection visit 2- Psychological and Tolerance Testing**
   The second in-person visit focused on psychological evaluation, involving 23 candidates from the first campaign and 25 from the second. Psychological assessments included three hours of questionnaires and a one-hour individual interview. The visit also included a centrifuge-based hypergravity tolerance test to determine individualized AG pre-syncopal times. Additional assessments included follow-up bloodwork (if needed), a chest X-ray, and a lower-limb venous Doppler ultrasound.
6. **Final Selection**
   After this phase, 24 participants and nine back-up candidates were selected for the study. Two participants withdrew for personal reasons before study start and were replaced by back-up candidates.

Reasons for exclusion

The main reasons for exclusion after the first selection visit were: anomalies in bone mass density (10/84), insufficient V̇O₂_peak_ (9/84), and bloodwork issues (6/84), including 3 subjects presenting a Factor V Leiden or prothrombin mutation, scoliosis (3/84), and excessive V̇O_2peak_ (2/84). Other reasons included a non-removable piercing that prevented MRI imaging, previously unreported personal dietary restrictions, difficult venous access, high or low BMI that had been misreported during the phone screening, use of hearing aid, ADHD, tobacco use, and inappropriate behavior. Additionally, six candidates cancelled their next planned visit.

Most candidates who were not included after completing their second selection visit were excluded for psychological reasons. Two others were excluded due to bloodwork anomalies identified during follow-up testing after the first visit. In total, twenty-four applicants and nine back-ups were selected.

Final selection

Twenty-four candidates and nine back-ups were ultimately selected to participate in the study. During the second campaign, two subjects withdrew before the study began: one due to a professional opportunity (a career advancement that was incompatible with a three-month absence), and the other four days before the start of the study due to family issues.

Figure legend

*Some candidates presented with more than one exclusion criterion,
which explains the discrepancy between the total number of excluded subjects
and the number of exclusion criteria.
