# Supplementary material for: Limited musculoskeletal benefits of artificial gravity combined with cycling during bed rest: Results from the BRACE study
Source: Exp Physiol. 2025 Nov 29;111(3):1181–90. doi: 10.1113/EP093145 (PMC12949100; doi:10.1113/EP093145)
Supplement: Supplementary file 2 — Detailed statistical output of comparisons and analyses [file EPH-111-1181-s001.pdf]

Variable	Comparison	F (df1, df2)	p-value	η^2p	M_{diff}	SE	95% CI	Cohen's D
Weight [kg]	Group	F(2,20) = 0.77	0.476	0.07	-	-	-	-
Total Thigh FFMV [L]	Group	F(2,20) = 10.9	< 0.001	0.52	-	-	-	-
	EX-AG vs C	-	< 0.001	-	0.79	0.17	[1.05, 3.66]	2.35
	EX-AG vs EX	-	0.092	-	0.39	0.17	[0.00, 2.29]	1.15
	EX vs C	-	0.087	-	0.41	0.18	[0.02, 2.39]	1.21
Left Anterior Thigh FFMV [L]	Group	F(2,20) = 22.4	< 0.001	0.69				
	EX-AG vs C	-	< 0.001	-	0.26	0.04	[1.78, 4.79]	3.28
	EX-AG vs EX	-	0.143	-	0.08	0.04	[-0.10, 2.11]	1.00
	EX vs C	-	< 0.001	-	0.18	0.04	[0.98, 3.58]	2.28
Left Posterior Thigh FFMV [L]	Group	F(2,20) = 4.2	0.031	0.29	-	-	-	-
	EX-AG vs C	-	0.041	-	0.14	0.05	[0.19, 2.50]	1.35
	EX-AG vs EX	-	0.100	-	0.11	0.05	[-0.02, 2.26]	1.12
	EX vs C	-	0.913	-	0.02	0.06	[-0.93, 1.38]	0.23
Right Anterior Thigh FFMV [L]	Group	F(2,20) = 12.4	< 0.001	0.55	-	-	-	-
	EX-AG vs C	-	< 0.001	-	0.24	0.05	[1.13, 3.77]	2.45
	EX-AG vs EX	-	0.263	-	0.08	0.05	[-0.28, 1.93]	0.83
	EX vs C	-	0.013	-	0.16	0.05	[0.43, 2.82]	1.63
Right Posterior FFMV [L]	Group	F(2,20) = 5.4	0.013	0.35	-	-	-	-
	EX-AG vs C	-	0.014	-	0.15	0.05	[0.41, 2.76]	1.59
	EX-AG vs EX	-	0.093	-	0.11	0.05	[0.00, 2.32]	1.16
	EX vs C	-	0.769	-	0.04	0.05	[-0.74, 1.58]	0.42
Total Thigh MFI [%]	Group	F(2,20) = 2.0	0.165	0.17	-	-	-	-
Mean Anterior MFI [%]	Group	F(2,20) = 2.2	0.134	0.18	-	-	-	-
Mean Posterior MFI [%]	Group	F(2,20) = 1.6	0.226	0.14	-	-	-	-
Left Anterior Thigh MFI [%]	Group	F(2,20) = 1.5	0.253	0.13	-	-	-	-
Left Posterior Thigh MFI [%]	Group	F(2,20) = 1.5	0.246	0.13	-	-	-	-
Right Anterior Thigh MFI [%]	Group	F(2,20) = 1.5	0.244	0.13	-	-	-	-
Right Posterior Thigh MFI [%]	Group	F(2,20) = 2.8	0.086	0.22	-	-	-	-
Weight-to-Muscle ratio [kg/L]	Group	F(2,20) = 11.3	< 0.001	0.53	-	-	-	-
	EX-AG vs C	-	< 0.001	-	-0.35	0.07	[-3.63, -1.04]	2.34
	EX-AG vs EX	-	0.299	-	-0.12	0.08	[-1.87, 0.31]	0.78
	EX vs C	-	0.016	-	-0.23	0.08	[-2.74, -0.38]	1.56
Fat Ratio [%]	Group	F(2,20) = 6.7	0.006	0.40	-	-	-	-
	EX-AG vs C	-	0.004	-	-2.73	0.75	[-3.04, -0.63]	1.83
	EX-AG vs EX	-	0.143	-	-1.48	0.75	[-2.09, 0.10]	1.00
	EX vs C	-	0.249	-	-1.25	0.76	[-1.93, 0.26]	0.84
Total Abdominal Adipose Tissue index [L/m^2]	Group	F(2,20) = 2.6	0.098	0.21	-	-	-	-
Abdominal Subcutaneous Adipose Tissue volume [L]	Group	F(2,20) = 2.5	0.105	0.20	-	-	-	-
Visceral Adipose Tissue volume [L]	Group	F(2,20) = 1.4	0.269	0.12	-	-	-	-
Visceral Adipose Tissue index [L/m^2]	Group	F(2,20) = 1.6	0.228	0.14	-	-	-	-
Visceral Adipose Tissue ratio [%]	Group	F(2,20) = 0.7	0.517	0.06	-	-	-	-
Liver Fat [%]	Group	F(2,17) = 0.5	0.462	0.09	-	-	-	-
Flexion, 30º/s (Nm)	Group	F(2,19) = 0.0	0.991	0.00	-	-	-	-
Extension, 30º/s (Nm)	Group	F(2,19) = 0.2	0.830	0.02	-	-	-	-
Flexion 90º/s (Nm)	Group	F(2,19) = 0.0	0.996	0.00	-	-	-	-
Extension 90º/s (Nm)	Group	F(2,19) = 0.9	0.405	0.09	-	-	-	-
Flexion, 180º/s (Nm)	Group	F(2,19) = 1.3	0.292	0.12	-	-	-	-
Extension, 180º/s (Nm)	Group	F(2,19) = 0.2	0.829	0.02	-	-	-	-
Flexion, 300º/s (Nm)	Group	F(2,18) = 0.7	0.494	0.08	-	-	-	-
Extension, 300º/s (Nm)	Group	F(2,18) = 1.8	0.193	0.17	-	-	-	-
